# Zika Virus Tissue and Blood Compartmentalization in Acute Infection of Rhesus Macaques

**DOI:** 10.1371/journal.pone.0171148

**Published:** 2017-01-31

**Authors:** Lark L. Coffey, Patricia A. Pesavento, Rebekah I. Keesler, Anil Singapuri, Jennifer Watanabe, Rie Watanabe, JoAnn Yee, Eliza Bliss-Moreau, Christina Cruzen, Kari L. Christe, J. Rachel Reader, Wilhelm von Morgenland, Anne M. Gibbons, A. Mark Allen, Jeff Linnen, Kui Gao, Eric Delwart, Graham Simmons, Mars Stone, Marion Lanteri, Sonia Bakkour, Michael Busch, John Morrison, Koen K. A. Van Rompay

**Affiliations:** 1 Department of Pathology, Microbiology and Immunology, School of Veterinary Medicine, University of California, Davis, California, United States of America; 2 California National Primate Research Center, University of California, Davis, California, United States of America; 3 Department of Psychology, University of California, Davis, California, United States of America; 4 Hologic, San Diego, California, United States of America; 5 Blood Systems Research Institute and University of California, San Francisco, California, United States of America; Tulane University, UNITED STATES

## Abstract

Animal models of Zika virus (ZIKV) are needed to better understand tropism and pathogenesis and to test candidate vaccines and therapies to curtail the pandemic. Humans and rhesus macaques possess similar fetal development and placental biology that is not shared between humans and rodents. We inoculated 2 non-pregnant rhesus macaques with a 2015 Brazilian ZIKV strain. Consistent with most human infections, the animals experienced no clinical disease but developed short-lived plasma viremias that cleared as neutralizing antibody developed. In 1 animal, viral RNA (vRNA) could be detected longer in whole blood than in plasma. Despite no major histopathologic changes, many adult tissues contained vRNA 14 days post-infection with highest levels in hemolymphatic tissues. These observations warrant further studies to investigate ZIKV persistence and its potential clinical implications for transmission via blood products or tissue and organ transplants.

## Introduction

Emerging mosquito-borne Zika virus (ZIKV, *Flaviviridae*, flavivirus) was first detected in Brazil in 2015 and has since spread to at least 60 countries in South and Central America, Oceania and Asia [1]. In 2015 and 2016, more than 4,800 imported cases have been reported in travelers returning to the continental United States. An additional 35,000 cases were reported in US Territories. Local mosquito-borne transmission in Florida was first documented in July 2016 with 210 local cases reported as of December 28, 2016 [2]. ZIKV is also transmitted sexually, via blood transfusion, and possibly organ transplants [3–6]. The World Health Organization declared the ongoing ZIKV outbreaks a public health emergency on February 1, 2016 [7] based on explosive geographic spread, clinical and epidemiological associations with microcephaly [8–11] and other neurological defects (reviewed in [12]), and lack of a licensed vaccine or specific therapies. ZIKV was historically considered a mild febrile illness with very little research dedicated to understanding infection dynamics and mechanisms of disease; therefore, very little is known about human ZIKV infections. Similar to other viral diseases, the availability of relevant animal models would be extremely useful for understanding human ZIKV infection dynamics and pathogenesis and to test intervention approaches such as vaccines, drugs, or other strategies with a goal of interrupting all modes of ZIKV transmission and treating infection. Such pre-clinical screening studies can guide clinical trials with the ultimate goal of ending the ZIKV pandemic. New murine models developed since March 2016 require immunodeficient mice [13–20], which is a major limitation. Since fetal development and placental biology of humans is more similar to non-human primates (NHP) than to rodents, NHP may better model adult infections and ZIKV-induced fetal neuropathogenesis than mice. Except for the first isolation of ZIKV in a rhesus macaque in Uganda in 1947 and experimental infection of several macaques with mouse-brain passaged ZIKV in the 1950s [21, 22], there were no published reports of ZIKV infection of non-human primates, and no animals had been experimentally inoculated with contemporary outbreak strains until 2016. In recent months, several studies using Asian or South-American lineage strains injected subcutaneously in rhesus or cynomolgus macaques showed infection marked by viremia, and that prior infection or immunization provided protection from subsequent challenge [23–26]. In this study, we monitored and euthanized 2 non-pregnant macaques 14 days post-intravenous inoculation of a 2015 Brazilian strain of ZIKV to characterize clinical disease, the time course of virus levels in plasma, whole blood, urine, and saliva. We also measured the kinetics of neutralizing and binding antibodies and ZIKV RNA distribution and histopathologic changes in tissues. We demonstrate that viral RNA could be detected longer in whole blood than in plasma and that despite no major histopathologic changes, many macaque tissues contained viral RNA 14 days post-infection.

## Results

### Experimental design

**Fig 1:**
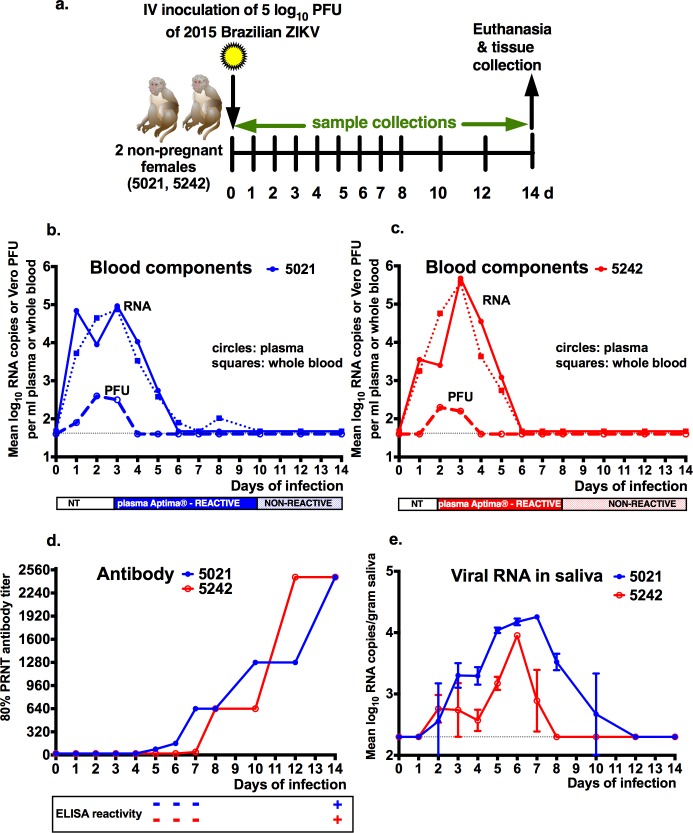
ZIKV infection of 2 adult macaques. a) Virus inoculation and sampling timeline. Two West Nile virus-antibody negative non-pregnant female macaques received an intravenous inoculation of 5.0 log_10_ PFU of a 2015 Brazilian ZIKV strain on day 0. Signs of clinical disease were recorded twice daily. Animals were anesthetized daily from 1 to 8 then on 10, 12 and 14 dpi for blood collection and euthanized for tissue collection 14 dpi. Plasma and whole blood ZIKV RNA and infectious virus levels and kinetics b) for animal 5021 and c) for animal 5242. ZIKV RNA levels in plasma (filled circles) and whole blood (filled squares) are reported in mean log_10_ RNA copies/ml and were assayed in duplicate. Infectious virus levels in plasma (open circles) were measured as Vero cell plaque forming units/ml. Colored bars under the graph show the period plasma tested ZIKV RNA reactive by the Aptima**®** assay. NT indicates ‘not tested. Dotted line shows limits of detection for both assays 1.6 log_10_ RNA copies or PFU per ml. d) Neutralizing and binding antibody kinetics and magnitude. ZIKV 80PRNT endpoint antibody titers are reported. The first plasma dilution tested was 1:20. The box shows the ZIKV IgG ELISA test results on plasma tested at a dilution of 1:50; plus and minus signs indicate positive or no reactivity, respectively. e) Saliva ZIKV RNA levels and kinetics. ZIKV RNA levels in saliva eluted from cotton swabs placed in the cheek of macaques, reported in mean log_10_ RNA copies/ml or gram, were assayed in triplicate with standard deviations noted. The dotted line shows the limit of detection, 2.3 log_10_ RNA copies/ml or gram.

**Fig 2:**
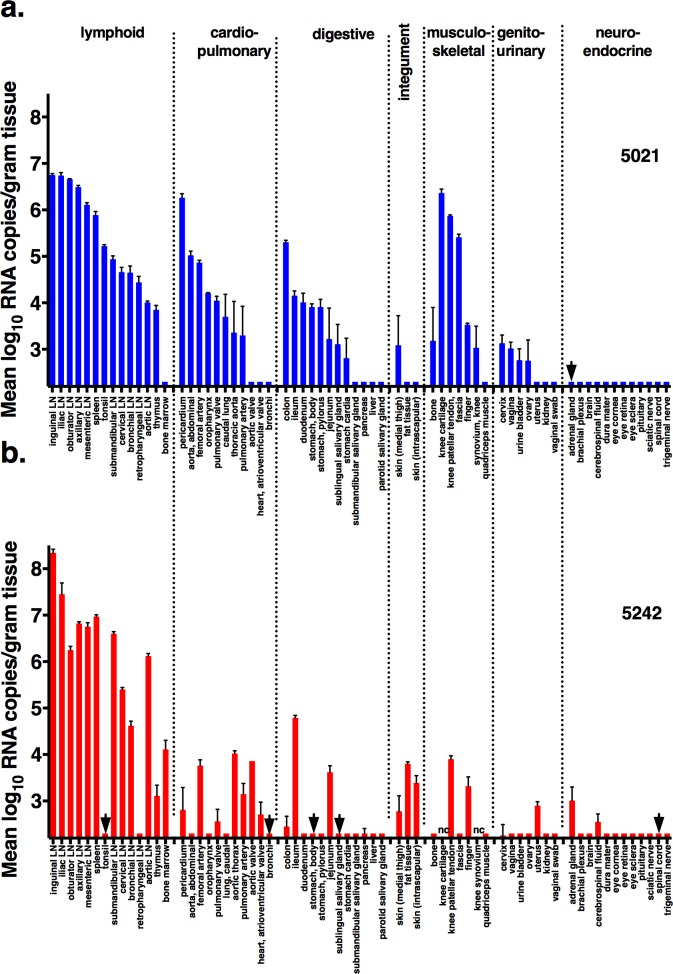
ZIKV tissue distribution in macaques. ZIKV RNA was measured by qRT-PCR assay in triplicate with standard deviations noted for **a)** animal 5021 and **b)** animal 5242. The limit of detection varied depending on the weight of tissue sampled and volume of MEM needed to homogenize to liquefaction, with a mean of 2.3 (range 1.4–4) log_10_ RNA copies per gram; non-reactive samples are reported at 2.0. log_10_ RNA copies per gram. Arrows indicate qRT-PCR negative samples that tested positive by the qualitative Aptima**®** assay. nc indicates not collected.

We inoculated 2 West Nile virus seronegative adult female rhesus macaques (numbers 5021 and 5242), 11–12 years of age, intravenously with 1 ml containing 5.0 log_10_ plaque-forming units (PFU; containing 7.8 log_10_ RNA copies) of a 2015 Brazilian ZIKV isolate. Intravenous inoculation was used to ensure infection and to mimic both mosquito feeding that is intra- and extra-venous [27] and transfusion-transmission. Animals were sedated for sample collection for the first 8 days, then sedated every subsequent 2 days, and euthanized at 14 days post-inoculation (dpi) for tissue collection (Figs 1 and 2).

### Clinical evaluations of ZIKV-inoculated rhesus macaques

Macaques were observed at least twice daily for clinical signs of disease including inappetence, stool quality, dehydration, diarrhea, and lethargy. Rectal temperature was collected at every sedation. No clinical signs, including fever, lethargy, or skin rash were observed in any of the animals. The 2 animals each exhibited ~10% weight loss from 0–14 dpi, likely due to frequent sedations that require overnight fasting. Complete blood counts and lymphocyte phenotyping data from sampling days showed minor changes such as a transient decrease of platelets and B lymphocytes during the first week of infection, and at approximately 1 week, a transient decrease of neutrophils and increase of monocytes and lymphocytes (reflected in all subpopulations of CD4+ T, CD8+T and NK cells, S1 and S2 Figs); these changes may be due to ZIKV infection as observed in humans (reviewed in [28]) and/or the frequent sedation schedule.

### Adult macaques clear plasma infectious virus and vRNA quickly and vRNA is detectable in whole blood for several days longer than plasma

Blood was sampled at indicated times (Fig 1A) and ZIKV vRNA levels were measured in duplicate and reported as mean log_10_ RNA copies per ml of plasma using an established quantitative reverse transcription polymerase chain reaction (qRT-PCR) [29] that was modified to use 22.7 ul of RNA. Both animals became vRNA positive within 1 day, with peak titers 3 days post-inoculation (dpi) at 5.0 or 5.7 log_10_ RNA copies/ml, and no vRNA was detected in plasma past day 7 (Fig 1B and 1C). Plasma tested by a more sensitive but qualitative Aptima**®** ZIKV assay developed by Hologic and licensed by the FDA [30] extended the period of vRNA detection by 1–2 days, to 8 or 10 dpi. We also tested longitudinal whole blood samples using the same qRT-PCR protocols as for plasma and detected ZIKV RNA up to 10 dpi in animal 5021, 3 days beyond detection of vRNA in plasma (Fig 1B). qRT-PCR positive plasma samples from the animals 1–8 dpi were also tested for infectious virus by plaque assay to determine titers in plaque forming units (PFU)/ml. Viremias peaked at 2.2 or 2.5 log_10_ PFU/ml 2 dpi and were not detectable after 4 dpi (Fig 1B and 1C). These results show that rhesus macaques intravenously inoculated with 5.0 log_10_ PFU of a 2015 Brazilian strain of ZIKV become infected and clear plasma of detectable infectious virus by 4 dpi, plasma of vRNA by 8 dpi, and whole blood of vRNA by 12 dpi.

#### Adult macaques produce neutralizing antibody coincident with clearance of plasma viremia

Neutralizing antibody titers in plasma were determined by endpoint 80% plaque reduction neutralization tests (PRNT80) using passage 2 of the ZIKV strain that was inoculated into macaques. Both animals developed PRNT80 titers of 640 by 8 dpi with detection beginning 4 or 7 dpi (Fig 1D). Titers in both animals rose incrementally from 8 dpi to a peak of 2,480 by the end of the experiment, 14 dpi. Thus, ZIKV inoculated macaques rapidly develop neutralizing antibody, coincident with the clearance of infectious virus from plasma. A qualitative ZIKV-specific IgG ELISA kit found that none of the animals had detectable IgG 7 dpi, but both had detectable IgG 14 dpi (Fig 1D). To monitor non-specific immune activation, plasma was tested for a panel of cytokines, chemokines, and growth factors via a multiplex microbead liquid array, but no consistent pattern was detected (S3 Fig), which is consistent with the relatively short, subclinical infection course of ZIKV in the macaques.

### ZIKV RNA is present in macaque urine and saliva

ZIKV vRNA levels were measured in urine and saliva in triplicate and reported as mean log_10_ RNA copies per ml of plasma using an the same quantitative reverse transcription polymerase chain reaction (qRT-PCR) as for plasma and whole blood and [29] 9.6 ul of RNA. ZIKV RNA was detected in genitourinary tissues from animal 5021 (Fig 2) and corroborated with vRNA in urine 6 and 8 dpi at 3.6 log_10_ RNA copies per ml on both days. ZIKV RNA was detected in saliva from both animals for 7 to 10 days and peaked at 4.2 log_10_ RNA copies/gram saliva (Fig 1E).

### ZIKV tissue distribution in adult macaques at 14 dpi is diffuse and variable

To assess vRNA levels and tropism in organs, macaque tissue samples were homogenized and ZIKV RNA levels were measured as for saliva and urine by qRT-PCR. Selected tissues that did not contain vRNA above the limit of the qRT-PCR assay were subsequently tested by the more sensitive Aptima**®** assay. (Fig 2, S1 Table). Many 14 dpi organs from both macaques contained detectable vRNA. Hemolymphatic tissues contained the highest levels, ranging from 3.1–8.3 log_10_ RNA copies/tissue with various lymph nodes and spleen the most highly positive, followed by cardiopulmonary, gastrointestinal, integument and genitourinary tissues. Kidneys, brain, eye and neuroendocrine tissues from both animals contained no detectable vRNA, except for the adrenal gland in both animals and cerebral spinal fluid and spinal cord of one animal. Selected tissues from different organ systems that varied in vRNA levels were chosen for titration. Titration data revealed no detectable infectious ZIKV in any tissues tested (axillary lymph node, heart, spleen, vagina, or uterus) above the 1.9 log_10_ PFU/ml tissue detection limit, although these tissues had vRNA levels >5 log_10_ RNA copies/ml. Since even the axillary lymph nodes that possessed among the highest (>6.5 log_10_ RNA copies/ml) vRNA levels across the sampled organs contained no detectable plaque competent ZIKV, other tissues with lower vRNA levels were not titrated by plaque assay. The high vRNA to PFU ratio is consistent with the disparity observed in plasma (Fig 1B and 1C).

### Adult ZIKV infected macaque tissues lack major histopathologic changes

Analyses of tissues collected from the 2 animals at necropsy 14 dpi when viremia or vRNA in plasma or whole blood was no longer detectable revealed no significant histopathologic changes (S2 Table).

## Discussion

After intravenous inoculation of ZIKV, the 2 rhesus macaques developed a short viremia, with plasma vRNA levels peaking at 5.7 log_10_ RNA copies/ml and remaining detectable to 7 dpi in plasma and 10 dpi in whole blood. Infectious virus was detectable in plasma to 4 dpi. Clearance of ZIKV from plasma coincided with the development of robust neutralizing antibody responses beginning 5 dpi that peaked at 2460 on 14 dpi when the experiment was terminated. The kinetics of vRNA in plasma, saliva, and urine in these macaques following intravenous inoculation with Brazilian ZIKV parallel data from other recent rhesus macaque studies [23–26] that used ZIKV isolates from areas other than Brazil and that were inoculated subcutaneously. In one of those studies [23], which used the same inoculation dose, qRT-PCR assay, and plasma volumes, plasma vRNA was detected intermittently at low levels to 21 dpi in non-pregnant animals; by contrast, neither of the adult animals in our study had detectable plasma vRNA at >0.7 log_10_ copies/ml, the Aptima**®** cut-off value, after 10 dpi. These differences may be due to a combination of inoculation route, methodological, viral, and host factors. Our intravenous inoculation likely resulted in antigen presentation to many lymph nodes simultaneously, possibly promoting faster innate immune responses with clearance of circulating vRNA, whereas subcutaneous inoculation used in the other study may have resulted in slower dissemination through the draining lymph node, as indicated by the broader window of peak viremia, 2 to 6 dpi, observed in that study. Another possible explanation for the disparity in length of plasma vRNA detection across the 2 macaque studies is differences in the 2015 Brazil and 2013 French Polynesia ZIKV genomes, although Asian lineage outbreak strains share >99% genome-wide nucleotide identity [31, 32]. Lastly, considering the small animal numbers in these pilot studies, other host factors including macaque genetics or other intra-animal differences, could have contributed. Control non-vaccinated macaques in two vaccine studies that were challenged with 3 or 6 log_10_ 2015 Puerto Rico ZIKV or a different Brazilian ZIKV strain than we used here (BeH815744, accession number KU365780) developed vRNA levels in plasma for 6 to 7 days that peaked 3 to 5 dpi at about 6 log_10_ copies/ml [25, 26], consistent with our observations. The pattern of transient viremia in all of the macaque studies and the absence of clinical disease including febrile responses parallels observations from humans where the majority of infections are sub-clinical [33–35].

This study is, to our knowledge, the first to compare ZIKV vRNA kinetics in plasma and whole blood of ZIKV-infected macaques. Although definitive conclusions cannot be made from a study involving only 2 animals, we observed that vRNA was detected in whole blood longer than in plasma in 1 of the macaques. In samples from ZIKV-infected humans, vRNA was sometimes detected in whole blood but not in plasma for up to 3 months, suggesting that vRNA compartmentalizes with red blood cells [6]. Further research is needed to understand the mechanisms underlying this difference. ZIKV vRNA may be in white blood cells, adhere to red blood cells, possibly mediated by ZIKV-specific antibody, and/or may be an after-effect of ZIKV infection of erythroid precursor cells in the bone marrow. Future studies in nonhuman primates can also investigate the clinical implications of ZIKV compartmentalization in whole blood, including to determine whether vRNA detection also represents infectious virus that could be transmitted through blood product transfusion.

Detection of ZIKV RNA in many 14 dpi tissues of the adult macaques shows that ZIKV exhibits a diffuse organ tropism, with highest tropism for hemolymphatic organs, similar to observations in other macaques [24] as well as many other blood-borne pathogens including other flaviviruses and HIV and SIV [36]. Since none of the animals in this study contained detectable vRNA in blood at necropsy 14 dpi, contamination of tissues by viremic blood is not likely, indicating that ZIKV levels were from resident cells in those tissues. For several tissues that were tested, no infectious virus could be isolated despite the high levels of vRNA. Considering the detection limit of the plaque assay (1.9 log_10_ PFU/ml tissue homogenate), the uncertainty how of PFU levels correlate with *in vivo* infectivity, and the potential inhibitory effects of neutralizing antibodies in tissue homogenates, further research in which macaques are inoculated with limiting dilutions of ZIKV will be useful to identify whether there is a threshold above which vRNA levels pose a significant risk of representing infectious virus. This can have significant implications for the safety of organ and tissue transplantation. Accordingly, further studies on the persistence and infectivity of ZIKV in organs and tissues over time are urgently needed to inform donor deferral and testing policies.

Of the 2 adult macaques in this study, only 1 had relatively low levels of vRNA in brain tissues and/or CSF, suggesting that in immunocompetent adult animals, the central nervous system (CNS) is not a major site of virus replication or that the window of infection is probably narrow and was not sampled in this study. The cerebellum of cynomolgus macaques peripherally inoculated with a Puerto Rican ZIKV strain contained detectable vRNA 5 dpi [24], 9 days earlier than the brains from rhesus macaques in this study were tested, supporting a short window of vRNA detection in CNS tissues. We acknowledge that our vRNA detection assay is least sensitive for brain tissue, as demonstrated by the spike experiment (S3 Table), possibly due to the high fat content that makes homogenization of brain tissue difficult. Overall, however, the observation of low levels or no detection of vRNA in brain or CSF in the adult animals in this pilot study is consistent with the fact that neither had signs of neurologic disease. In human adults, neurologic disease is relatively rare. The ZIKV outbreak in French Polynesia and other countries has been associated with Guillain-Barré syndrome [37, 38], and rare case reports have described encephalitis, meningoencephalitis, and myelitis [39]. The latter 2 conditions were accompanied by detection of ZIKV RNA in CSF of 2 patients [40, 41], suggesting that infection of the central nervous system is directly responsible for neurologic ZIKV disease, although confounding host immunologic factors have not been excluded. Direct virus-mediated neurologic disease has been observed for the related flavivirus dengue virus [42]. Further studies with larger animal numbers will be required to determine if ZIKV infection can induce similar neurologic disease in adult macaques.

In summary, in this pilot study, we demonstrate that intravenous infection of adult rhesus macaques with a Brazilian ZIKV strain resulted in a short plasma viremia marked by infectious virus and vRNA, with more prolonged detection of vRNA in whole blood and a diffuse vRNA distribution in many tissues at 2 weeks post-infection. These data underscore the need for further studies in the macaque model to evaluate the kinetics of persistence and infectivity of ZIKV in different fluids, tissues, and organs, so that if a significant risk of transmission exists, various interventions can be developed to reduce such risks in all settings, including possible interventions such as active or passive immunization, antivirals, or pathogen reduction techniques to treat blood products or tissue and organ transplants.

## Materials and Methods

### Animals, care, use and sample collection

The animals in this study were healthy adult female Indian-origin rhesus macaques (*Macaca mulatta*), 11 to 13 years of age, from the type D retrovirus-free, Herpes B virus free, SIV-free and simian lymphocyte tropic virus type 1-free colony, and confirmed to be antibody negative for West Nile virus (WNV) using a simian WNV ELISA (Xpress Bio). Animals were not screened for dengue virus antibody given the absence of local circulation in California. Both animals were negative for the MHC Class I Mamu-A*01 and A*08 alleles and positive for MamuB*01. Animals were housed and all experimental procedures were performed at the California National Primate Research Center (CNPRC), a facility accredited by the Association for Assessment and Accreditation of Laboratory Animal Care International (AAALAC). All animal care was performed in compliance with the Guide for the Care and Use of Laboratory Animals provided by the Institute for Laboratory Animal Research (2011). The macaques were housed indoor in stainless steel cages (Lab Product, Inc.) and were exposed to a 12 hour light/dark cycle, 65–75°F, and 30–70% room humidity. Animals had free access to water and received commercial chow (high protein diet, Ralston Purina Co.) and fresh fruit supplements. The study was approved by the Institutional Animal Care and Use Committee of the University of California, Davis. When necessary, macaques were immobilized with ketamine HCl (Parke-Davis) at approximately 10 mg/kg and injected intramuscularly after overnight fasting. Blood samples were collected using venipuncture. Cerebrospinal fluid was collected via a cervical spinal tap. Urine was collected on days of sedation via cystocentesis. Animals were euthanized with an overdose of pentobarbital, followed by necropsy with extensive tissue collection.

### ZIKV inoculations

Zika virus (strain Zika virus/H.sapiens-tc/BRA/2015/Brazil_SPH2015; genbank accession number KU321639.1) isolated from the plasma of a human in Brazil in 2015 was passaged once on Vero cells and then titrated by Vero cell plaque assay. The inoculum was adjusted to 5.0 log_10_ PFU (corresponding to 7.8 log_10_ RNA) in 1 ml of RPMI-1640 medium and injected intravenously in the saphenous vein. Inocula were back-titrated by plaque assay to verify the administered dose. The inoculum tested negative for mycoplasma contamination by deep sequencing (not shown).

### vRNA isolation from plasma, fluids, and tissues

EDTA-anticoagulated whole blood was collected and processed immediately. A 200 μl aliquot was removed and frozen immediately at -80°C. The rest of the blood was centrifuged for 10 m at 800 g to separate plasma from cells. The plasma was spun an additional 10 m at 800 g to further remove cells, and aliquots were immediately frozen at -80°C. The cellular blood fraction was diluted with PBS and layered on lymphocyte separation medium (MP Biomedicals) and spun for 30 m at 800 g to isolate peripheral blood mononuclear cells that were washed and then cryopreserved for future analyses. Tissues were added to 2.0 ml round bottom tubes holding a 5 mm steel bead and 250 ul Dulbecco’s modified eagle medium (DMEM). Tubes were weighed and tissue weights were determined by subtracting the mass of the tube, bead, and medium without the tissue. Tissues were homogenized for 2 m at 30 shakes/second (s) in a Mixer Mill (Qiagen). The homogenate was centrifuged for 2 m at 14,000 g to clarify the supernatant. If liquefaction of tissues was incomplete, samples were homogenized for an additional 2 m at 30 shakes/s and if that second step failed to achieve liquefaction, an additional 250 ul of DMEM was added, the tube was weighed again, and the sample was re-homogenized. To ensure homogenization did not markedly reduce vRNA quantities, select tissues from non-infected macaques were spiked with ZIKV and homogenized. The same ZIKV stock, ‘spike input’, was added to DMEM alone or tissues from non-ZIKV inoculated macaques and homogenized. Mean vRNA levels were compared between the samples with and without the tissue. ZIKV RNA levels from homogenized spiked tissue samples showed reduced vRNA levels from 0.7–3.5 log_10_ RNA copies compared to spike input, with a mean loss of 1.7 log_10_ RNA copies (S3 Table). RNA was extracted from 140 ul of plasma, CSF, urine, or homogenized tissue supernatants using the viral RNA mini kit (Qiagen). All RNA extracts were eluted in 60 ul of DEPC-treated water for storage at -80°C prior to quantification. Levels of vRNA in samples are expressed as mean log_10_ vRNA copies per gram of tissue or ml.

### vRNA isolation from saliva and vaginal swabs

Saliva was collected from sedated animals by placing 2 Weck-cel sponges (Beaver Visitec) in the cheek pouches for several minutes. A dry microbial culture swab was used to collect vaginal secretions. All swabs were frozen at -80°C. On testing day, swabs were thawed and incubated for 30m at room temperature in 250 μl DMEM and 12 4 mm glass beads at the bottom of the tube. Samples were vortexed for 10 seconds and then centrifuged for 5–8 m at 500 g to pull the liquid out of the swab. vRNA was then extracted from the liquid as for other liquid samples. Viral levels from oral swabs are expressed as mean log_10_ vRNA copies per gram saliva.

### vRNA quantitation by quantitative real time polymerase chain reaction (qRT-PCR)

For urine, saliva and organ samples, Zika virus RNA was measured in triplicate by reverse transcription qRT-PCR on an Applied Biosystems ViiA 7 machine using the Taqman Fast Virus 1-Step Master Mix (Thermo) and published primers and probe from Lanciotti *et al*. (ZIKV 1086, ZIKV 1162c, and ZIKV 1107-FAM) with 9.6 ul of RNA. In an effort to improve sensitivity by increasing sample volume tested, the plasma and whole blood samples were tested in duplicate using 22.7 ul of RNA (of 60 ul total) in a 50 ul RT-PCR reaction volume with a 2x reaction buffer and enzyme (SuperScript III Platinum One-Step qRT-PCR Kit from Thermo). For each 96-well plate testing urine, saliva, and organ samples, a standard curve was generated from serial dilutions of the inocula vRNA of known concentration. To determine concentration, the Brazil 2015 stock strain was quantified relative to a standard curve of the French Polynesia 2013 stock strain. The latter was quantified both by a computational method based on maximum-likelihood estimation for copy, and also relative to a quantified virus standard obtained from the European Virus Archive (Zika standard #1, H/PF/2013 inactivated). Both quantitation methods yielded the same copy number value for the French Polynesia 2013 stock strain. For plasma and whole blood, standards were derived from spiked quantified vRNA diluted in human plasma that were included on every plate. MEM diluent processed alongside samples was used as a negative control. The sensitivity of the assay is detailed in Stone et. al. [43]. The limit of detection of viral RNA copies from liquid samples was 1.6 (plasma and whole blood) or 2.3 log_10_ copies/ml (saliva and urine). For organs, the limit of detection varied depending on the weight of tissue sampled and volume of DMEM needed to homogenize to liquefaction, with a mean of 2.3 (range 1.4–4) log_10_ RNA copies. For selected samples, the Hologic Aptima® ZIKV assay [30] was used on a higher volume (0.5 ml) of plasma or tissue homogenate. Aptima® is a qualitative *in vitro* assay that detects vRNA on a fully automated Panther® system and is currently approved for clinical use. All steps are performed in one tube with an internal control and a of detection of 0.7 log_10_ copies/ml.

### Viral quantification by plaque assay

Vero cells starting at passage 5 from the American Type Culture Collection were used for virus titrations by plaque assay. Confluent 6- or 12-well Vero plates were inoculated with 250 or 125 μl, respectively, of ten-fold dilutions of macaque plasma, cerebral spinal fluid, urine, or tissue homogenates in MEM supplemented with 10% fetal bovine serum and allowed to absorb at 37°C for 1h. When plasma samples were viscous and clumped undiluted, the first dilution tested was 1:10. After incubation, each cell monolayer was overlaid with 2 (12-well plates) or 4 ml (6-well plates) 0.8% agar (liquefied 10% agar, ultrapure agarose [Invitrogen] diluted in 42°C MEM) and allowed to solidify. The plates were incubated at 37°C for 8 days. Cell monolayers were then fixed with 4% formalin for 30m, agar plugs were removed, and 0.025% gentian violet (Sigma) in 30% ethanol was overlaid in each well to stain viable cells. Viral titers were recorded as the reciprocal of the highest dilution where plaques are noted. The limit of detection of the assay was 1.9 log_10_ PFU/ml for 12-well plates and 1.6 log_10_ PFU/ml for 6-well plates.

### Neutralizing antibody quantification by plaque reduction neutralization test (PRNT)

Endpoint 80% PRNT titers were determined in macaque plasma. Plasma samples were heated to 56°C for 30 m to inactivate complement, serially 2-fold diluted starting at 1:10 (1:20 final virus:plasma dilution) in 150 μl MEM with 10% FBS and then incubated for 1h at 37°C with approximately 80 PFU of ZIKV stock from a second Vero passage. After 1h, virus-antibody or virus-only mixtures were overlaid on confluent monolayers, and the plaques developed under agar overlays were counted after 8 d under gentian violet staining, as for plaque assays. Dilutions of plasma that caused a >80% reduction in the number of plaques, as compared with negative controls (MEM only), were considered positive. The reciprocal of the highest dilution of plasma, indicated as the final virus-serum dilution, that inhibited at least 80% of plaques is reported as the antibody titer.

### Zika antibody detection by ELISA

Plasma samples from selected time points were tested at a 1:50 dilution for ZIKV antibody using a commercially available ELISA (Xpress Bio), according to manufacturer’s instructions. The target antigen is a recombinant NS-1 protein based on the sequence of an African lineage ZIKV strain (Zika virus/H.sapiens-tc/UGA/1947/Uganda_MR766; genbank accession number KU955594.1) produced in human HEK 293 cells. Reactivity is determined through parallel testing of each sample on wells coated with ZIKV antigen versus a negative control antigen. Since the conjugate is anti-IgG, the assay detects mainly ZIKV-specific IgG.

### Measurement of non-specific immune markers in fluids

Cytokines, chemokines and growth factors were measured in EDTA-anticoagulated plasma and amniotic fluid samples using the Cytokine Monkey Magnetic 29-Plex Panel for Luminex™ Platform (Thermofisher Scientific), according to the manufacturer’s instructions

### Complete blood count measurements

Complete blood counts (CBC) were performed on EDTA-anticoagulated blood using a Pentra 60C+ electronic cell counter (Horiba Diagnostics) with a manual differential cell count on standard blood smear with Wright-Giemsa stain (Harleco, EM Scientific). To define T and B lymphocytes and NK cells, 4-color flow cytometry techniques, consisting of a single tube containing antibodies to CD3, CD4, CD8 and CD20, were used and samples were analyzed on a FACSCalibur flow cytometer, as described previously [44], with exception that CD4 was detected using FITC-conjugated anti-CD4 antibody clone L200, and that all antibodies were from BD Biosciences.

### Histopathologic analyses using hemotoxylin and eosin

Macaque tissues were placed in 10% buffered formalin followed by standard processing and then paraffin embedding, sectioning, and hematoxylin and eosin staining.

## Supporting Information

S1 Fig Complete Blood Count data from ZIKV-infected macaques. Percentages of neutrophils, lymphocytes and monocytes are expressed with white blood cells in the denominator. wbc refers to white blood cells.
(PDF)

S2 Fig Lymphocyte phenotyping in ZIKV-infected macaques. Flow cytometry was used to measure CD4+CD3+ T lymphocytes, CD8+CD3+ T lymphocytes, CD8+CD3- NK cells and CD20+CD3- B lymphocytes.
(PDF)

S3 Fig Cytokines, chemokines and growth factors in macaque plasma. Cytokine markers consisted of interleukin (IL)-1β, IL-1RA (interleukin-1 receptor antagonist), IL-2, -4, -5, -6,-10, -12, -15 and -17, GCSF (granulocyte colony-stimulating factor), GM-CSF (granulocyte macrophage colony-stimulating factor), IFN-γ (interferon gamma), IP-10 (interferon gamma-induced protein 10), and TNF-α (tumor necrosis factor alpha). Chemokines consisted of eotaxin, IL-8, MCP-1 (monocyte chemoattractant protein 1), MDC (macrophage-derived chemokine), MIF (macrophage migration inhibitory factor), MIG (monokine induced by gamma interferon), MIP-1α (macrophage inhibitory protein 1-alpha), MIP-1β, I-TAC (Interferon-inducible T-cell alpha chemoattractant), RANTES (regulated on activation, normal T cell expressed and secreted). Growth factors included EGF (epidermal growth factor), FGF-2 (basic growth factor), HGF (hepatocyte growth factor) and VEGF (vascular endothelial growth factor). For some markers, the range of values observed in 6 healthy uninfected adult macaques (4 males, 2 females) is indicated by a horizontal line.
(PDF)

S1 Table Aptima® Testing of qRT-PCR negative macaque tissues. All tissues in the table tested negative by qRT-PCR and were subsequently re-tested using the more sensitive qualitative Aptima® assay. Reactive indicates detection of vRNA, nonreactive means no vRNA detected.
(PDF)

S2 Table Pathological Findings in ZIKV-infected adult macaques. The minus sign indicates no histologic changes; N/A indicates tissue not available or not applicable.
(PDF)

S3 Table Spike experiment to assess reduction in ZIKV RNA levels after tissue homogenization. The same ZIKV stock, ‘spike input’, was added to MEM alone or tissues from non-ZIKV inoculated macaques and homogenized. Mean vRNA levels were compared between the samples with and without the tissue and showed that spiked vRNA levels were reduced in homogenized tubes containing macaque tissues.
(PDF)
