# Supplementary material for: Zika Virus Tissue and Blood Compartmentalization in Acute Infection of Rhesus Macaques
Source: PLoS One. 2017 Jan 31;12(1):e0171148. doi: 10.1371/journal.pone.0171148 (PMC5283740; doi:10.1371/journal.pone.0171148)

S1 Fig. Complete Blood Count data from ZIKV-infected macaques. Percentages of neutrophils, lymphocytes and monocytes are expressed with white blood cells in the denominator. wbc refers to white blood cells.

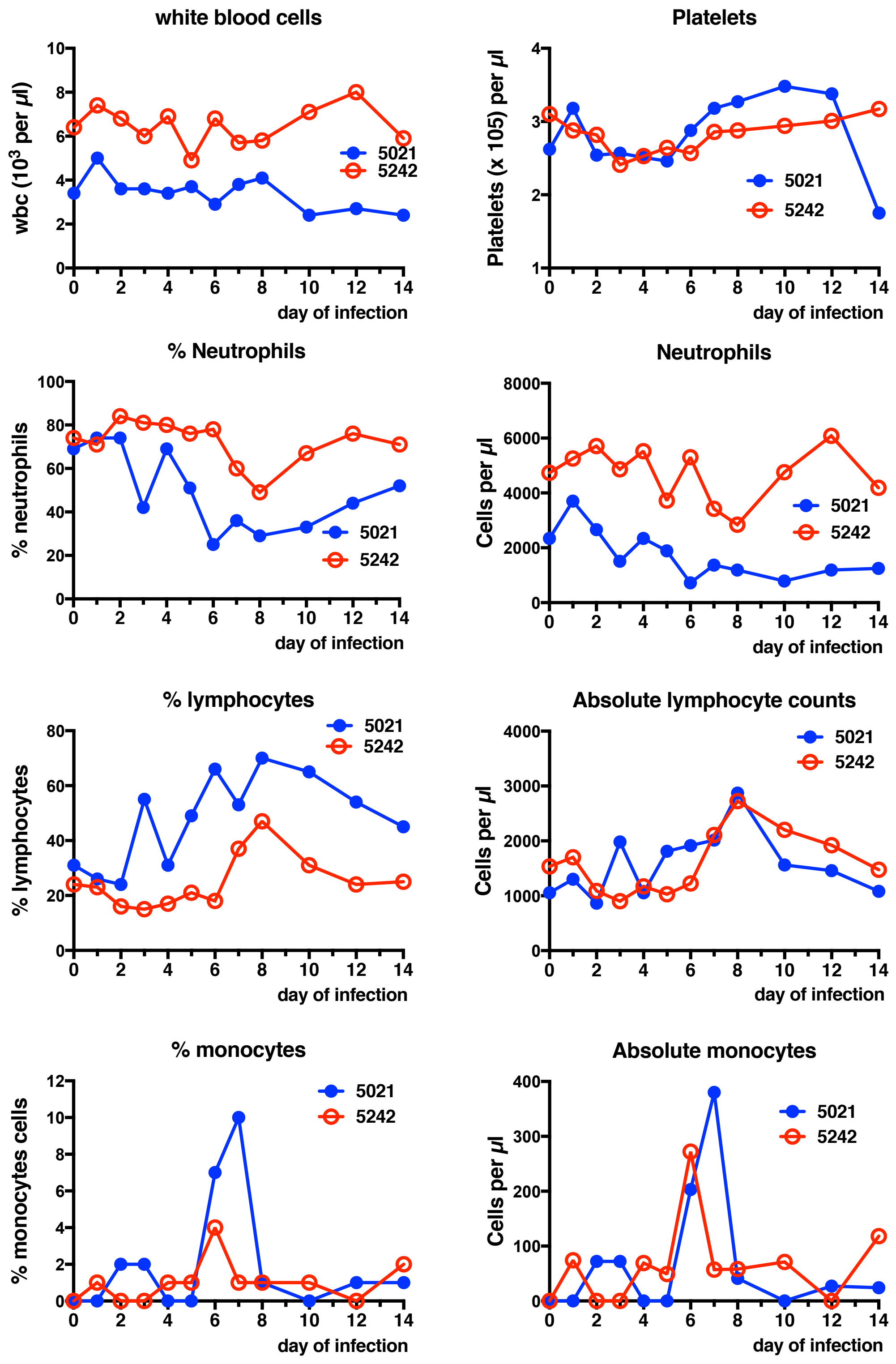

Supplement: S1 Fig — Percentages of neutrophils, lymphocytes and monocytes are expressed with white blood cells in the denominator. wbc refers to white blood cells. (PDF) [file pone.0171148.s001.pdf]
