# Supplementary material for: Zika Virus Tissue and Blood Compartmentalization in Acute Infection of Rhesus Macaques
Source: PLoS One. 2017 Jan 31;12(1):e0171148. doi: 10.1371/journal.pone.0171148 (PMC5283740; doi:10.1371/journal.pone.0171148)

S2 Fig. Lymphocyte phenotyping in ZIKV-infected macaques. Flow cytometry was used to measure CD4+CD3+ T lymphocytes, CD8+CD3+ T lymphocytes, CD8+CD3- NK cells and CD20+CD3- B lymphocytes.

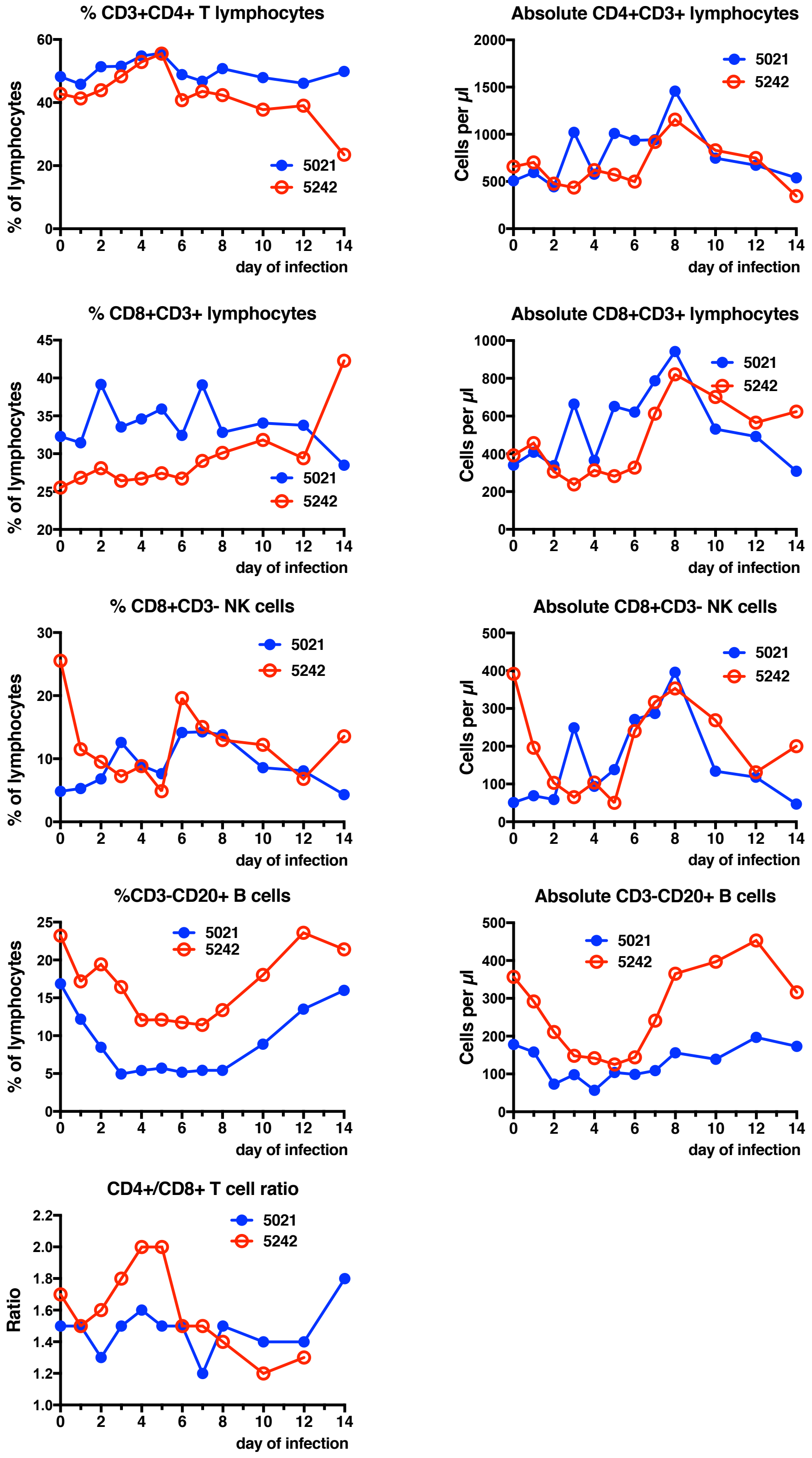

Supplement: S2 Fig — Flow cytometry was used to measure CD4+CD3+ T lymphocytes, CD8+CD3+ T lymphocytes, CD8+CD3- NK cells and CD20+CD3- B lymphocytes. (PDF) [file pone.0171148.s002.pdf]
