# Supplementary material for: Zika Virus Tissue and Blood Compartmentalization in Acute Infection of Rhesus Macaques
Source: PLoS One. 2017 Jan 31;12(1):e0171148. doi: 10.1371/journal.pone.0171148 (PMC5283740; doi:10.1371/journal.pone.0171148)
Supplement: S3 Fig — Cytokine markers consisted of interleukin (IL)-1β, IL-1RA (interleukin-1 receptor antagonist), IL-2, -4, -5, -6,-10, -12, -15 and -17, GCSF (granulocyte colony-stimulating factor), GM-CSF (granulocyte macrophage colony-stimulating factor), IFN-γ (interferon gamma), IP-10 (interferon gamma-induced protein 10), and TNF-α (tumor necrosis factor alpha). Chemokines consisted of eotaxin, IL-8, MCP-1 (monocyte chemoattractant protein 1), MDC (macrophage-derived chemokine), MIF (macrophage migration inhibitory factor), MIG (monokine induced by gamma interferon), MIP-1α (macrophage inhibitory protein 1-alpha), MIP-1β, I-TAC (Interferon-inducible T-cell alpha chemoattractant), RANTES (regulated on activation, normal T cell expressed and secreted). Growth factors included EGF (epidermal growth factor), FGF-2 (basic growth factor), HGF (hepatocyte growth factor) and VEGF (vascular endothelial growth factor). For some markers, the range of values observed in 6 healthy uninfected adult macaques (4 males, 2 females) is indicated by a horizontal line. (PDF) [file pone.0171148.s003.pdf]

S3 Fig. Cytokines, chemokines and growth factors in macaque plasma. Cytokine markers consisted of interleukin (IL)-1β, IL-1RA (interleukin-1 receptor antagonist), IL-2, -4, -5, -6, -10, -12, -15 and -17, GCSF (granulocyte colony-stimulating factor), GM-CSF (granulocyte macrophage colony-stimulating factor), IFN-γ (interferon gamma), IP-10 (interferon gamma-induced protein 10), and TNF-α (tumor necrosis factor alpha). Chemokines consisted of eotaxin, IL-8, MCP-1 (monocyte chemoattractant protein 1), MDC (macrophage-derived chemokine), MIF (macrophage migration inhibitory factor), MIG (monokine induced by gamma interferon), MIP-1α (macrophage inhibitory protein 1-alpha), MIP-1β, I-TAC (Interferon-inducible T-cell alpha chemoattractant), RANTES (regulated on activation, normal T cell expressed and secreted). Growth factors included EGF (epidermal growth factor), FGF-2 (basic growth factor), HGF (hepatocyte growth factor) and VEGF (vascular endothelial growth factor). For some markers, the range of values observed in 6 healthy uninfected adult macaques (4 males, 2 females) is indicated by a horizontal line.

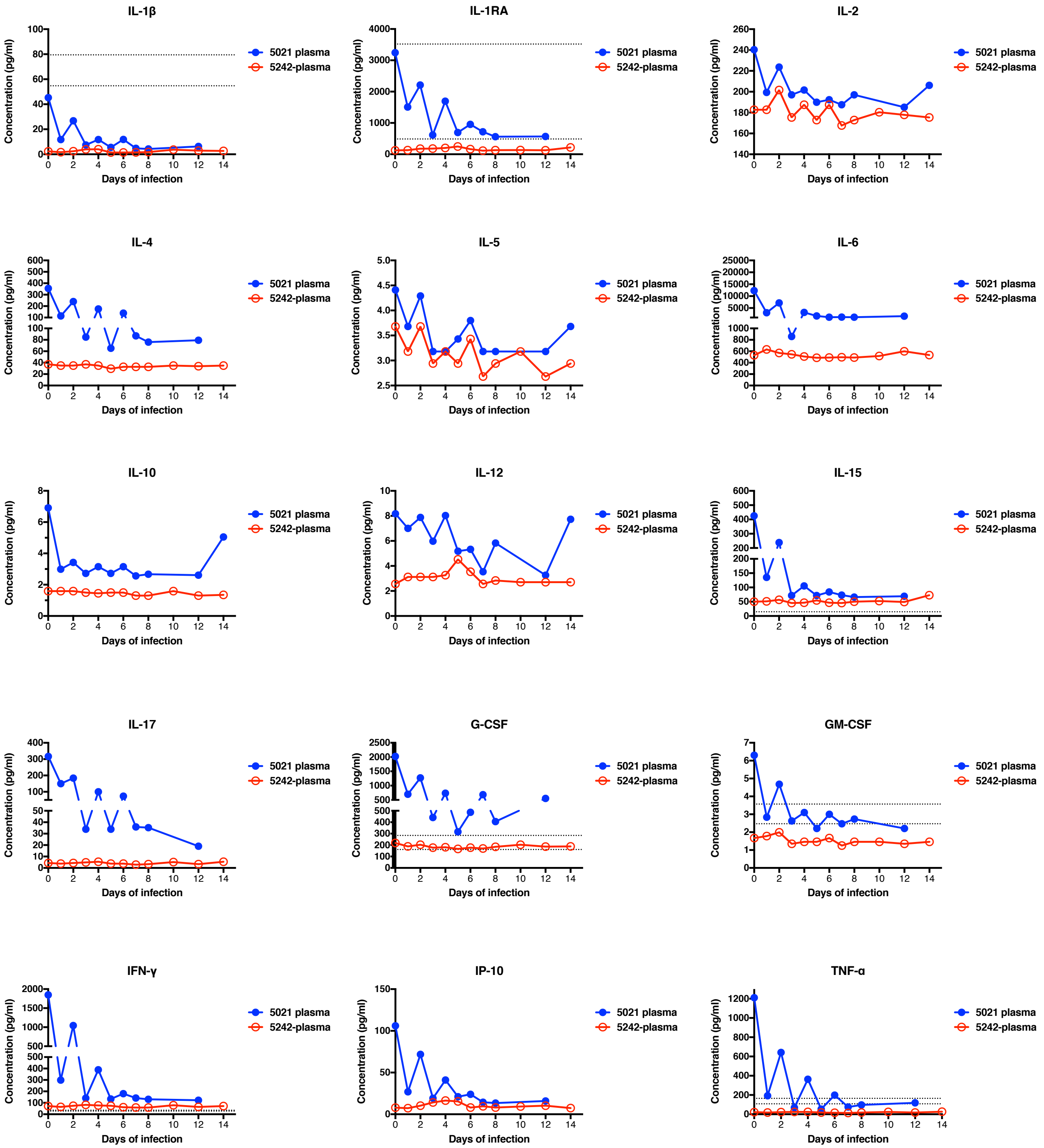
IL-1β
IL-1RA
IL-2
IL-4
IL-5
IL-6
IL-10
IL-12
IL-15
IL-17
G-CSF
GM-CSF
IFN-γ
IP-10
TNF-α
Concentration (pg/ml)
Days of infection
5021 plasma
5242-plasma

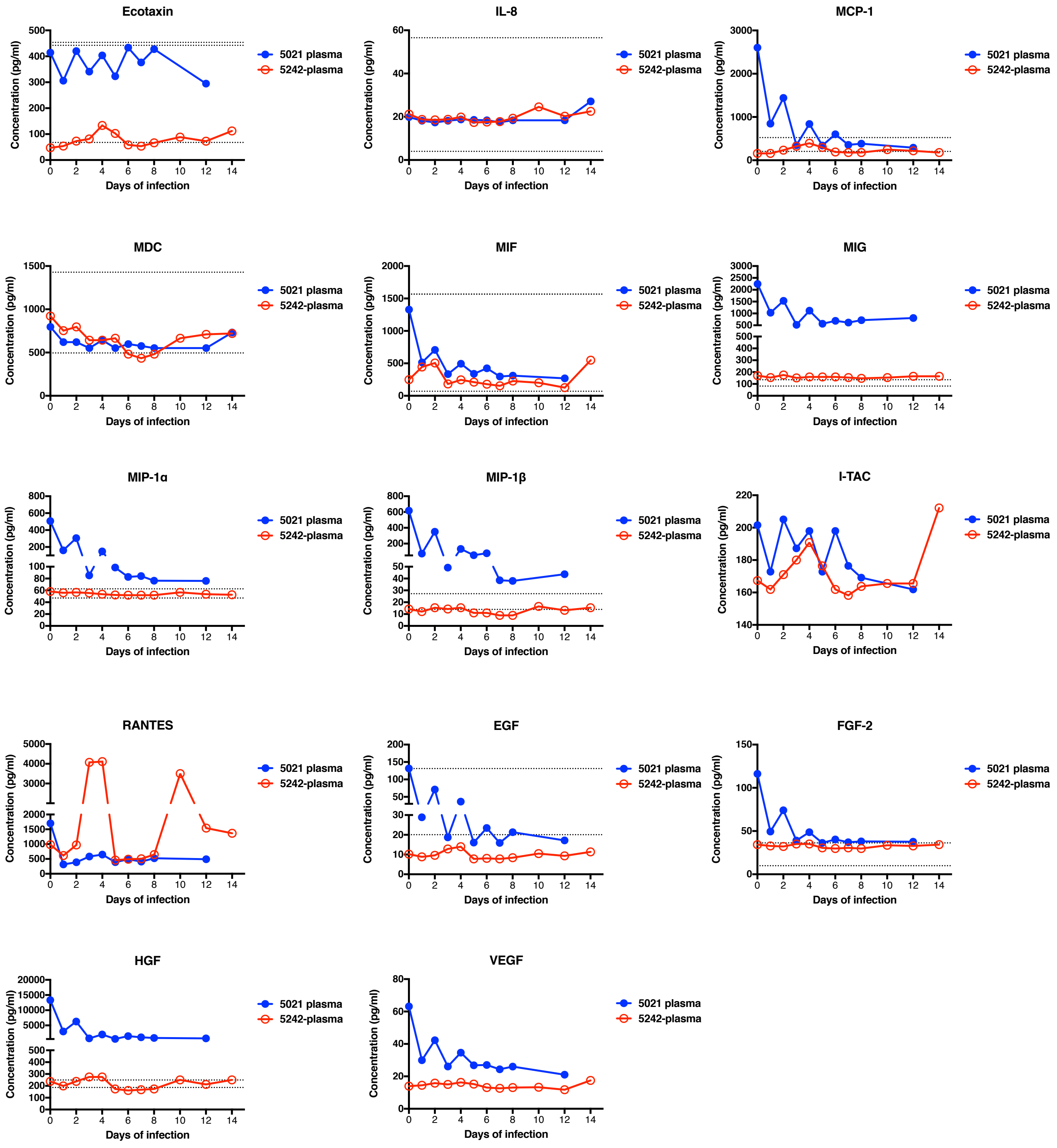

Ecotaxin
IL-8
MCP-1
MDC
MIF
MIG
MIP-1α
MIP-1β
I-TAC
RANTES
EGF
FGF-2
HGF
VEGF
Concentration (pg/ml)
Days of infection
5021 plasma
5242-plasma
