# Supplementary material for: Zika Virus Tissue and Blood Compartmentalization in Acute Infection of Rhesus Macaques
Source: PLoS One. 2017 Jan 31;12(1):e0171148. doi: 10.1371/journal.pone.0171148 (PMC5283740; doi:10.1371/journal.pone.0171148)
Supplement: S1 Table — All tissues in the table tested negative by qRT-PCR and were subsequently re-tested using the more sensitive qualitative Aptima® assay. Reactive indicates detection of vRNA, nonreactive means no vRNA detected. (PDF) [file pone.0171148.s004.pdf]

S1 Table. Aptima® testing of qRT-PCR negative macaque tissues. All tissues in the table tested negative by qRT-PCR and were subsequently re-tested using the more sensitive qualitative Aptima® assay. Reactive indicates detection of vRNA, nonreactive means no vRNA detected.

Macaque	Tissue	Aptima ZIKV Result
5021	submandibular salivary gland	Nonreactive
	pancreas	Nonreactive
	parotid salivary gland	Nonreactive
	aortic valve	Nonreactive
	bronchi	Nonreactive
	skin (intrascapular)	Nonreactive
	dura mater	Nonreactive
	eye cornea	Nonreactive
	eye retina	Nonreactive
	eye sclera	Nonreactive
	adrenal gland	Reactive
	brachial plexus	Nonreactive
	brain	Nonreactive
	pituitary	Nonreactive
	sciatic nerve	Nonreactive
	spinal cord	Nonreactive
	trigeminal nerve	Nonreactive
	brain	Nonreactive
5242	tonsil	Reactive
	retropharyngeal lymph node	Nonreactive
	sublingual salivary gland	Reactive
	parotid salivary gland	Nonreactive
	lung, caudal	Nonreactive
	aortic valve	Nonreactive
	bronchi	Reactive
	duodenum	Nonreactive
	stomach, body	Reactive
	stomach, pylorus	Nonreactive
	stomach, cardia	Nonreactive
	fascia	Nonreactive
	quadriceps muscle	Nonreactive
	kidney	Nonreactive
	urine bladder	Nonreactive
	ovary	Nonreactive
	brachial plexus	Nonreactive
	dura mater	Nonreactive
	eye cornea	Nonreactive
	eye retina	Nonreactive
	eye sclera	Nonreactive
	pituitary	Nonreactive
	sciatic nerve	Nonreactive
	spinal cord	Reactive
	trigeminal nerve	Nonreactive
