# Supplementary material for: Zika Virus Tissue and Blood Compartmentalization in Acute Infection of Rhesus Macaques
Source: PLoS One. 2017 Jan 31;12(1):e0171148. doi: 10.1371/journal.pone.0171148 (PMC5283740; doi:10.1371/journal.pone.0171148)
Supplement: S2 Table — The minus sign indicates no histologic changes; N/A indicates tissue not available or not applicable. (PDF) [file pone.0171148.s005.pdf]

S2 Table. Pathological findings in ZIKV-infected adult macaques. The minus sign indicates no histologic changes; N/A indicates tissue not available or not applicable.

Tissue	Animal number	
	**5021**	**5242**
**Heart**	-	-
**Spleen**	Mild lymphoid hyperplasia	Mild lymphoid hyperplasia
**Lung**	Mild multifocal pneumoconiosis	-
**Kidney**	-	-
**Liver**	Mild Ito cell hyperplasia	Mild Ito cell hyperplasia
**Gall bladder**	-	-
**Stomach**	Moderate lymphoplasmacytic gastritis with intraglandular spiral bacteria (presumed Helicobacter)	Intraglandular spiral bacteria (presumed Helicobacter) without inflammation
**Duodenum**	-	-
**Jejunum**	-	-
**Ileum**	-	-
**Colon**	-	-
**Lymph nodes**		
**- axillary**	-	Mild lymphoid hyperplasia
**- inguinal**	Mild lymphoid hyperplasia	Mild lymphoid hyperplasia
**- mesenteric**	Mild lymphoid hyperplasia	Mild lymphoid hyperplasia
**- iliac**	Mild lymphoid hyperplasia	Mild lymphoid hyperplasia

**- obturator**	Mild lymphoid hyperplasia	Mild lymphoid hyperplasia
**- cervical**	-	-
**- submandibular**	N/A	-
**- bronchial**	-	N/A
**Salivary glands**		
**- parotid**	-	-
**- sublingual**	N/A	-
**- submandibular**	-	Rare periductular lymphocytes
**Thoracic Aorta**	-	-
**Pericardium**	-	-
**Knee joint**	-	-
**Bone**	-	-
**Bone marrow**	-	-
**Uterus**	N/A	-
**Cervix**	N/A	-
**Urinary bladder**	-	-
**Adrenal gland**	-	-
**Eye**	-	-
**Cervical spinal cord**	-	-
