# Supplementary material for: Zika Virus Tissue and Blood Compartmentalization in Acute Infection of Rhesus Macaques
Source: PLoS One. 2017 Jan 31;12(1):e0171148. doi: 10.1371/journal.pone.0171148 (PMC5283740; doi:10.1371/journal.pone.0171148)
Supplement: S3 Table — The same ZIKV stock, ‘spike input’, was added to MEM alone or tissues from non-ZIKV inoculated macaques and homogenized. Mean vRNA levels were compared between the samples with and without the tissue and showed that spiked vRNA levels were reduced in homogenized tubes containing macaque tissues. (PDF) [file pone.0171148.s006.pdf]

S3 Table. Spike experiment to assess reduction in ZIKV RNA levels after tissue homogenization. The same ZIKV stock, 'spike input', was added to MEM alone or tissues from non-ZIKV inoculated macaques and homogenized. Mean vRNA levels were compared between the samples with and without the tissue and showed that spiked vRNA levels were reduced in homogenized tubes containing macaque tissues.

		**mean $\log_{10}$ ZIKV RNA copies/gram measured after homogenization**	**$\log_{10}$ ZIKV RNA copies/gram post-homogenization loss**
	non-homogenized ZIKV spike input	9.6	n/a
**non-ZIKV infected macaque tissues spiked with ZIKV**	bladder	8.2	1.4
	brain	6.0	3.6
	liver	8.3	1.3
	muscle	8.8	0.8
